# Natural mutations in key NLS amino acids regulate nucleoplasmic shuttling and replication efficiency in PRRSV

**DOI:** 10.3389/fmicb.2025.1587634

**Published:** 2025-07-04

**Authors:** Xianchang Zhu, Yang Xia, Qian Lei, Yu Gan, Shenghai Jiang, Lian Huang, Qihu Wen, Wei Fu, Bo Zhang, Yi Zhang, Shanshan Xie, Jida Li

**Affiliations:** ^1^Institute of Zoonosis, College of Public Health, Zunyi Medical University, Zunyi, Guizhou, China; ^2^Southwest Guizhou Vocational and Technical College for Nationalities, Xingyi, Guizhou, China; ^3^Department of Preventive Health Care, Yunan District People's Hospital, Yunfu, Guangdong, China; ^4^College of Basic Medicine, Zunyi Medical University, Zunyi, Guizhou, China; ^5^College of Veterinary Medicine, Henan Agricultural University, Zhengzhou, Henan, China; ^6^Key Laboratory of Maternal & Child Health and Exposure Science of Guizhou Higher Education Institutes, Zunyi, Guizhou, China

**Keywords:** PRRSV, nucleocapsid protein, NLS, NTR, nuclear-mass shuttle

## Abstract

Highly pathogenic porcine reproductive and respiratory syndrome virus (HP-PRRSV) is a mutant strain of the classic porcine reproductive and respiratory syndrome virus (PRRSV) characterized by high morbidity and mortality rates. Epidemiological analysis revealed a natural mutation and stable inheritance of amino acid 46 (41-PGKKNKK-47 mutated to 41-PGKKNRK-47) in the nuclear localization signal or sequence (NLS) region of the N protein of HP-PRRSV. In this study, we showed that the nucleoplasmic shuttling of the HP-PRRSV N protein was associated with a higher efficiency of viral replication than that of the classical PRRSV. The nuclear transporter receptors KPNB1, KPNA1, KPNA2, KPNA6, and KPNA7 were involved in the nuclear import of the N protein. Additionally, the mRNA expression levels of KPNB1 and KPNA1 differed between the two strains after infecting the Marc-145 cells with these strains. The viral replication efficiency also decreased when expression levels of KPNA1 and/or KPNB1 were lowered. Finally, protein binding simulation and kinetic assay showed that the mutation of key amino acid 46 in the NLS region altered the binding mode and kinetics of the N proteins to KPNA1 and KPNB1. This study elucidates, for the first time, the reasons for the enhanced nucleoplasmic shuttling and replication efficiency of HP-PRRSV from the perspective of protein entry into the nucleus. It also provides a foundational reference for the prevention and control of PRRSV.

## 1 Introduction

Porcine reproductive and respiratory syndrome virus (PRRSV) is a single-stranded positive-stranded RNA virus belonging to the genus Arterivirus in the family Arteriviridae of the order Nidovirales. It is characterized by a genome size of about 15 kb, primarily known for causing porcine reproductive and respiratory syndrome (PRRS). Arterivirus is a single-stranded positive-stranded RNA virus that causes PRRS and is marked by reproductive disorders in sows and respiratory diseases affecting pigs of all age groups (Pan et al., 2023; Contreras-Luna et al., 2022). Since its first discovery, PRRSV has been mutating and recombining and the emergence of highly pathogenic porcine reproductive and respiratory syndrome virus (HP-PRRSV), which is presented by relatively high morbidity and lethality rates compared to classical PRRSV, has posed a major challenge for the pig industry, resulting in even greater economic losses (Du et al., 2021). Although researchers continue to invest a great deal of research in this field (Yang et al., 2024; Sun et al., 2024; Bai et al., 2024), the underlying cause for the enhanced pathogenicity of HP-PRRSV remains obscure. This issue has become one of the key problems in swine disease research requiring immediate mitigation.

Nucleolar localization signal (NLS) is a specific amino acid sequence found on the macromolecules that mediate their binding to nuclear entry transporter receptors, allowing them to enter the nucleus through the nuclear pore complex (Cross et al., 2024). The nuclear translocation signaling pathway mediated by Importin alpha/beta [Karyopherin alpha ( KPNA)/Karyopherin beta (KPNB)] is the most classical nuclear import pathway (Moraes et al., 2024; Pörschke et al., 2023; Lange et al., 2020). This process is marked by its increased selectivity and precision regulated by a combination of factors. The NLS, Importin α, and Importin β have been shown to play key roles in this process. These include two nuclear localization sequences (NLS1 and NLS2) situated on the N protein of PRRSV, with the NLS2 sequence (41-PGKKNKK-47) facilitating the entry of N proteins into the nucleus (Rowland and Yoo, 2003). Moreover, importin α can be categorized into seven isoforms including KPNA1 and KPNA7 which can co-exist within the same cell (Yamada et al., 2024; Tsimbalyuk et al., 2022; Oostdyk et al., 2019). Moreover, Importin β has been found to have at least 20 members among which Importin β1 (KPNB1) is the first widely studied isoform in current research and is known to play an essential role in various cellular processes (Ma et al., 2024; Fang et al., 2024). Numerous studies have demonstrated a strong correlation between NLS and the mechanisms of viral infection. In addition, the mutations in the NLS sequence present in the viral nucleoprotein considerably affect the pathogenicity and replication capacity of the virus. For example, when lysine mutates to arginine at position 214 in the NLS2 region of influenza, a virus nucleoprotein (NP) weakens the binding of NLS2 to Importin α, reducing the capacity of the nuclear import (Wu et al., 2017). Moreover, protein mutations of the newcastle disease virus (NDV) matrix (M) in lysine and arginine, at position 247 in the hybrid region (247–266) of the NLS and nuclear export signal (NES) can alter the efficiency of nucleoplasmic shuttling of the M protein. This change subsequently affects the assembly and the release of the virus, exerting a significant impact on the viral pathogenicity of the virus (Peng et al., 2022). Taking into account the epidemiological findings obtained in our laboratory, the NLS2 sequence of HP-PRRSV exhibits a consistent mutation at position 46 (K → R), where the sequence 41-PGKKNK-47 has mutated to 41-PGKKNRK-47 in the N protein when compared to classical PRRSV; this mutation has been stably inherited (Li et al., 2012). Therefore, we propose that the high pathogenicity of HP-PRRSV compared to that of classic PRRSV is associated with mutations in key amino acids in the NLS region of the N protein.

Although some studies have found that the enhanced immune evasion ability of HP-PRRSV is associated with the mutations at this locus (Fan et al., 2015), there is no relevant research elucidating whether this stems from the changes in the NLS-mediated nucleation mechanism and its impact on the replication ability of HP-PRRSV. In this study, we investigated the effects of mutations in the NLS region of HP-PRRSV N protein for the first time from the perspective of protein nucleation. This will provide a new perspective for elucidating the mechanisms underlying the enhancement of HP-PRRSV pathogenicity and serve as an essential reference for screening new antiviral drug targets.

## 2 Materials and methods

### 2.1 Cells and virus

Marc-145 cells were purchased from iCell Bioscience Inc. (Shanghai, China), and JXA1 (representative strain of HP-PRRSV) was purchased from the Chinese Center for Disease Control and Prevention. VR2332 (a classical strain of PRRSV) was purchased from Boehringer Ingelheim Bio-Products Co. Ltd. (Taizhou, China), and JXA1-R (a vaccine strain of HP-PRRSV) was purchased from Qilu Animal Health Products Co., Ltd. (Jinan, China). Marc-145 cells were melted in a water bath at 37°C and added with Dulbecco's modified Eagle medium (DMEM, Gibco, Waltham, Massachusetts, USA) containing 10% fetal bovine serum (FBS, Deary Tech, Changchun, China). The cells were cultured at 37°C and 5% CO_2_. The viral suspension preparation process is as follows: thaw the three viral strains stored at −80°C in an ice bath, dilute them with DMEM at a 1:10 ratio, and inoculate them into Marc-145 cell culture flasks (25 cm^2^) containing cells at ~80% confluence. Incubate at 37°C for 1 h, then add DMEM supplemented with 2% FBS to a final volume of 5 mL. Incubate for ~72 h, until most cells exhibit cytopathic effect (CPE). Centrifuge at 4°C and 3,000 × g for 15 min, then collect the supernatant. Filter the supernatant through a 0.22 μm membrane filter, aliquot into cryopreservation tubes, and store at −80°C for future use. Ensure that the same batch of viral solution is used for the same experiment to maintain experimental comparability.

### 2.2 Virus titration

The Marc-145 cells grown in 96-well plates were infected with a 10-fold serial dilution of the virus. The supernatant was incubated at 37°C for 2 h and then replaced with DMEM in 2% FBS. Three to five days after transfection, the cells showed a cytopathic effect of aggregation and contraction. The virus titration was calculated according to the Reed-Muench method and expressed as log_10_ TCID_50_/mL.

### 2.3 Western blot analysis

The cells were collected and the total protein was extracted by adding RAPI lysate (Beyotime, Shanghai, China). The protein concentration was measured according to the instructions of the BCA kit (Coolaber, Beijing, China) and mixed with 5 × protein sampling buffer (Solarbio, Beijing, China) for metal bath denaturation. The target proteins were separated by SDS-PAGE gel, transferred to PVDF membrane (Bio-Rad, Hercules, CA, USA), and closed by adding a rapid closure solution. The cells were incubated overnight at 4°C with the indicated primary antibodies (PRRSV N protein monoclonal antibody obtained from Guangzhou Qianxun Biotechnology Co., Ltd. (Guangzhou, China) Thereafter, the cells were incubated at 4°C overnight and were then incubated with HRP-conjugated secondary antibodies (Proteintech, Wuhan, China) for 2 h at room temperature. Subsequently, luminescent signals were detected with ECL. Moreover, the signals were detected using the ECL western blot substrate (Thermo Fisher Scientific, Waltham, MA, USA). Finally, the bands were analyzed using Image J software (https://imagej.net/) for grayscale quantification.

### 2.4 Cell immunofluorescence assay

The cells were removed, washed with PBS, and fixed with 4% paraformaldehyde (Biosharp, Hefei, China) at room temperature. Following this, the cells were washed, added with Triton X-100 (Sangon Biotech Co., Ltd., Shanghai, China) for 45 min at room temperature, and then washed. The cells were closed with 5% BSA (Coolaber, Beijing, China) at room temperature and then incubated overnight at 4°C with the indicated primary antibody (PRRSV N protein). After washing, 5% of BSA (Coolaber, Beijing, China) was added and incubated at room temperature with the indicated primary antibody (PRRSV N protein) overnight at 4°C. The sample was incubated with IgG Alexa Fluor^®^ 488 (Proteintech) at room temperature for 2 h after washing. Then the cells were incubated with DAPI (Solarbio, Beijing, China) at room temperature, washed, and photographed under a fluorescence microscope. Nuclear efficiency % = (number of viral particles in the nucleus / total number of viral particles) × 100%. At least three independent experiments must be conducted before performing a differential analysis.

### 2.5 Quantitative real-time PCR analysis

The cells were collected, RNA was extracted according to the Trizol method, and the reverse transcription reaction was performed according to the instructions for the PrimeScript™ RT reagent Kit (Takara, Kusatsu, Japan), followed by the instructions for the 2 × SG Fast qPCR Master Mix (Takara, Kusatsu, Japan). The relative expression levels of the indicated genes were normalized to the level of GAPDH expression using the 2^−ΔΔCT^ method. The primers used for qPCR are listed in Table 1. The N protein-F sequence is positioned at nucleotides 14957 to 14982, while the N protein-R sequence is located at nucleotides 15044 to 15068 within the JXA1 holosequence.

**Table 1:** qPCR primers.

**Primer name**	**Primer sequence (5'-3')**
GAPDH-F	TGCCAAGTACGATGACATCAAGAAGG
GAPDH-R	GAAGAGTGGGTGTCGCTGTTGAAG
N protein-F	CCATTTCCCTCTAGCGACTGAAGATG
N protein-R	ACAAGTTCCAGCACCCTGATTGAAG
KPNB1-F	TGCATCAGTAGACAGAACTTCGGTTAG
KPNB1-R	GTGAGTGAGGCGGTATCCACATTC
KPNA1-F	TATCAGCCAAGAGTGTGACGCATTC
KPNA1-R	ACAGACATCAGACGGTATCCATCCC
KPNA2-F	TCTTCCTACCTTAGTTCGGCTCCTG
KPNA2-R	TTCGTTCATTCGGACCGTCAGTAAG
KPNA3-F	GGCATTCTCCTCGTTCGCACTAG
KPNA3-R	GGAGCGGTCGTCAACTACAGTTC
KPNA4-F	GGCGGACAACGAGAAACTGGAC
KPNA4-R	CTGGAAGGCACTGTTAGCATCACC
KPNA5-F	CAGTGACCCAGATGTGTTAGCAGAC
KPNA5-R	GTTCCACCAATCTTCGACAGACTCC
KPNA6-F	GGGCTGTGGTCCTTCCTCTCTC
KPNA6-R	ACACTCTCACTCCAACCTCCTCTG
KPNA7-F	CACAACAAGCCCTCCATCCAGAAG
KPNA7-R	GAGGCAAGACATCGTAGGCAAGC

### 2.6 RNA interference

Suzhou GenePharma Co., Ltd. (Suzhou, China) was commissioned to design and synthesize targeted Small interfering RNAs (siRNAs). These included the KPNA1 (siKPNA1:5-GCUGAUGCAUAAUGAUUUAUTT-3), KPNB1 (siKPNB1:5-GCUCAAACCACUAGUUUAUATT-3), and non-targeting control (control) siRNA (siNC:5-UUCUCCGAACGUGUUCACGUTT-3). The samples were collected and tested at specific times after the transfection operations were conducted according to the company's instruction manual.

### 2.7 Protein binding simulation prediction

The following data was retrieved from the NCBI website: KPNB1 (accession number: XM_008012949.2), KPNA1 (accession number: XM_007985606.2), HP-PRRSV N protein (accession number: EF112445.1), and classic PRRSV N protein (accession number: U87392.3). The proteins were obtained in three dimensions using UniPort (https://www.uniprot.org/) and Alphafold3 (https://alphafoldserver.com/welcome) and HP-PRRSV N protein was structurally optimized (energy-minimizing treatment) after amino acid point mutation based on the classic PRRSV N protein. Proteins were docked using the specialized HDOCKv1.1 tool (http://huanglab.phys.hust.edu.cn/software/hdocklite/). In addition, molecular docking and conformational scoring were performed using the iterative scoring function ITScore-PP, where negative scores indicated binding and larger absolute values indicated better binding ability. The maximum number of output conformers for docking was set to 100, and the top 10 conformers were scored. The binding complex structure with optimal binding was obtained. Gromacs 2018 (https://www.gromacs.org/) was chosen as the kinetic simulation software and Amber-ildn (https://github.com/yunhuige/ff99sb-ildn-nmr) as the protein and small molecule force field. TIP3P water was added to the system using the TIP3P model to create a water box with a size of 10 × 10 × 10 nm^3^ and ions were added to achieve an automatic system balance.

### 2.8 Protein expression purification and bio-layer interferometry assay

The above four proteins were purified using the *E. coli* prokaryotic expression system and pET-28a (+) was selected as the expression vector. To ensure the accurate and efficient expression of the target proteins by the constructed plasmids and to facilitate subsequent protein purification, His and Sumo tags were added to the N-terminus. After overnight incubation, the four successfully constructed plasmids were transferred to DH5α (Takara, Kusatsu, Japan) for sequencing and identification. Bacteria exhibiting accurate sequences were cultured in an expanded culture and transferred to Rosstta (Beyotime, Shanghai, China) after plasmid extraction using the SanPrep Column Plasmid DNA Mini-Extraction Kit (Sangon Biotech Co., Ltd., Shanghai, China). After plasmid extraction with (Sangon Biotech Co., Ltd., Shanghai, China), the plasmid was transferred to Rosstta (Beyotime, Shanghai, China) and incubated in shaking bacteria until the OD_600_ value was 0.6–0.8, then IPTG (Coolaber, Beijing, China) was added to induce protein expression. Using the His tag carried by the expressed protein, Ni-NTA Sepharose Resin (Sangon Biotech Co., Ltd., Shanghai, China) was selected and purified according to the instructions. The purified protein was stored at −80°C for subsequent Bio-Layer Interferometry (BLI) detection of the binding kinetics of PRRSV N protein to KPNA1 and KPNB1 before and after mutations in the key amino acids of the NLS.

### 2.9 Statistical analysis

SPSS 29.0 software (https://www.ibm.com/products/spss-statistics) was used for statistical analyses. The data were expressed as $\bar{X}\;\pm \;SEM$. One-way ANOVA was employed to analyze the data with uniform variances. LSD was used for pairwise comparisons. Kruskal Wallis test was used to analyze the data with unequal variances. A significance level of α = 0.05 was used. Statistical significance was set at *p* < 0.05. GraphPad Prism 9 software (https://www.graphpad.com/) was used to draw the graphs.

## 3 Results

### 3.1 JXA1 and JXA1-R replicate more efficiently than VR2332

**Figure 1:**
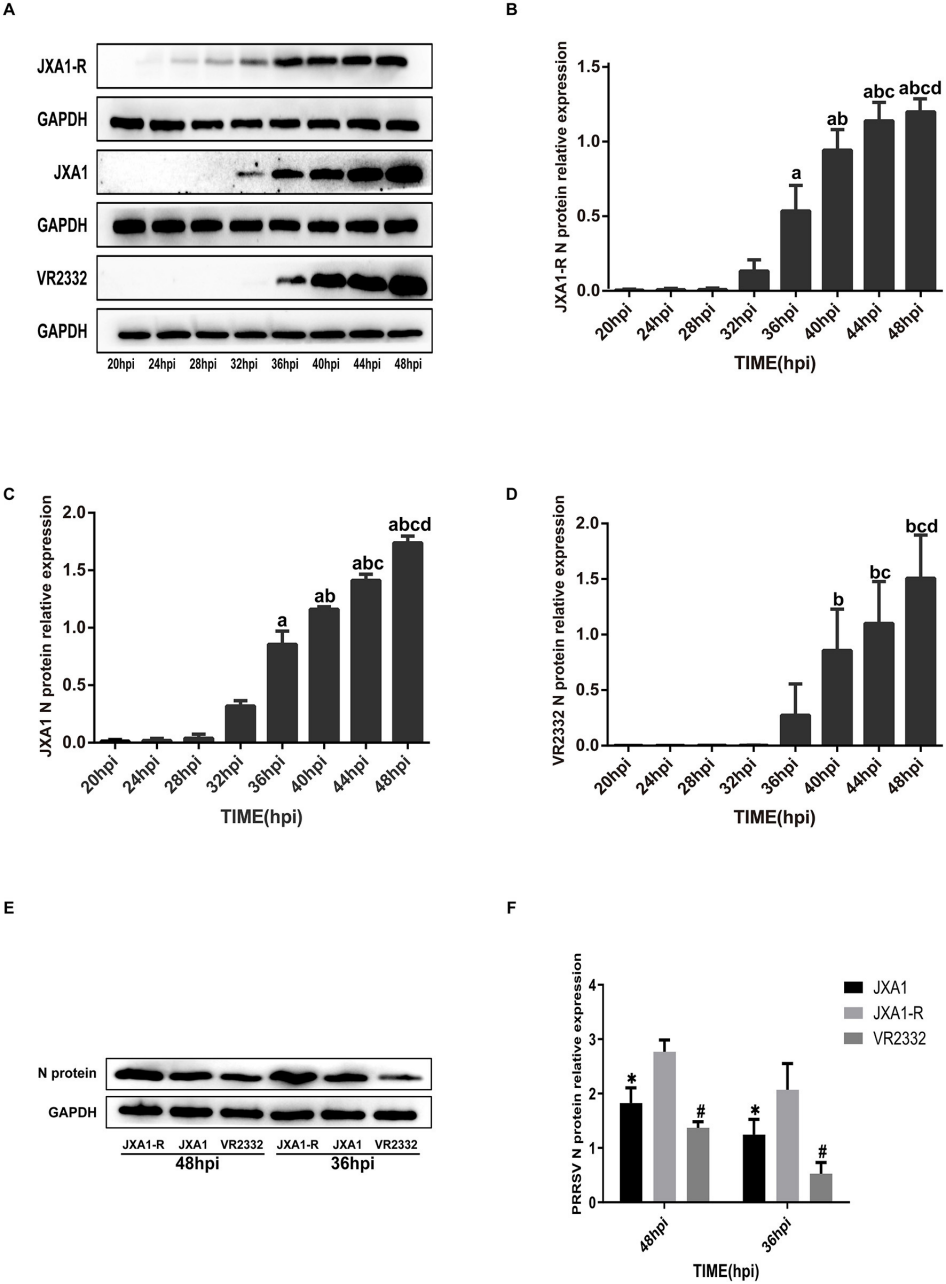
Differential expression analysis of PRRSV N protein. **(A–D)** a indicates comparison with 32 hpi, *p* < 0.001; b indicates comparison with 36 hpi, *p* < 0.001; c indicates comparison with 40 hpi, *p* < 0.001; d indicates comparison with 44 hpi, *p* < 0.001. **(E, F)** “*” indicates comparison with JXA1-R at the same time point, *p* < 0.01; “#” indicates comparison with JXA1 at the same time point, *p* < 0.01.

To evaluate the replication efficiency of the three strains JXA1, JXA1-R, and VR2332, we infected Marc-145 cells (MOI = 0.1) and monitored the expression of N protein at different time points. Western blot analysis showed that the expression levels of the three N protein strains showed an increasing trend as the infection time increased (Figure 1A). However, the expression levels of the N protein differed among different strains. Specifically, the expression levels of JXA1-R, JXA1, and VR2332 started at 24 hpi, 32 hpi, and 36 hpi, respectively (Figures 1B–D). The comparison of N protein expressions at 36 hpi and 48 hpi among the three strains revealed the same trend: JXA1-R>JXA1>VR2332 (Figures 1E, F).

### 3.2 The number of nuclear localizations and overall expression levels of JXA1 and JXA1-R was more than that of VR2332

**Figure 2:**
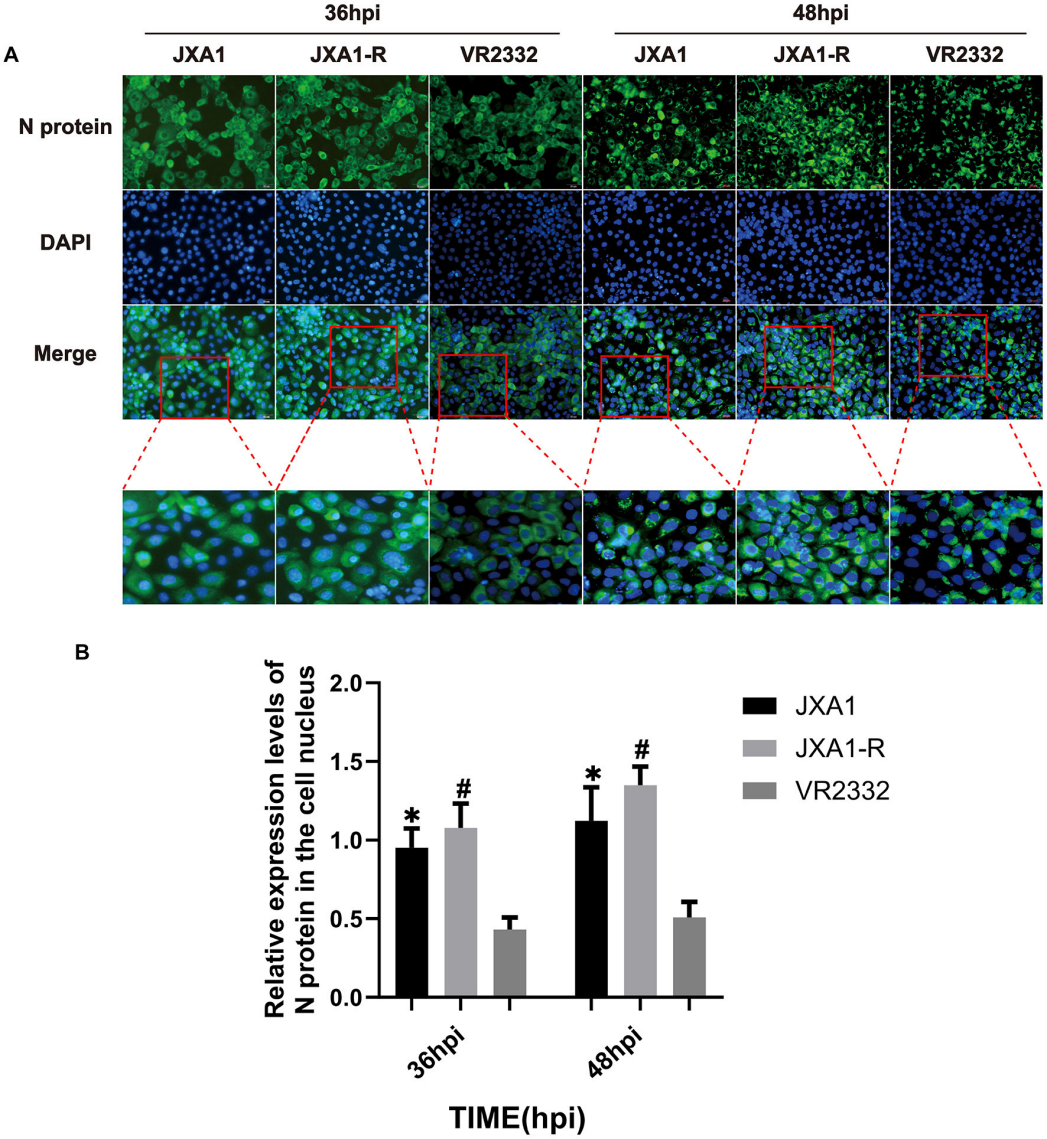
Nucleoplasmic shuttling and expression levels of PRRSV N protein. **(A)** Nucleoplasmic shuttling and expression of N protein in Marc-145 cells infected with three strains. **(B)** Differential analysis of the nuclear entry ratio of N protein in Marc-145 cells infected with three strains. “*” indicates a comparison between JXA1 and VR2332 at the same infection time, *p* < 0.01; “#” indicates a comparison between JXA1-R and VR2332 at the same infection time, *p* < 0.01.

To investigate whether there is a difference in the nucleoplasmic shuttling of the three strains of N protein, we infected the Marc-145 cells (MOI = 0.1) and monitored the localization and the amount of N protein at different time points. Cellular immunofluorescence experiments showed that the number of N proteins entering the nucleus and their expression levels increased gradually in the three strains as the infection time prolonged. JXA1-R showed N protein expression at 8 hpi, with the N protein localization in the nucleus observed at 12 hpi, 28 hpi, 32 hpi, 36 hpi, 44 hpi, and 48 hpi, especially prominent at 36 hpi and 48 hpi (Supplementary Figure S1). Similarly, JXA1 demonstrated the N protein expression starting from 8 hpi, and N protein localization in the nucleus was observed at 12 hpi, 20 hpi, 28 hpi, 36 hpi, 44 hpi, and 48 hpi, especially at 36 hpi and 48 hpi (Supplementary Figure S2). N protein expression for VR2332 started at 12 hpi, with a notable localization of the N protein in the nucleus observed at 16 hpi, 20 hpi, 32 hpi, 36 hpi, 44 hpi, and 48 hpi. In addition, the localization of the N protein in the nucleus was observed, again noted at 36 hpi and 48 hpi (Supplementary Figure S3). A comparative analysis showed that the number of nuclear localizations and overall expression of N proteins in JXA1-R and JXA1 were more than that in VR2332 at the same infection time (Figures 2A, B), revealing differences in the nucleoplasmic shuttling behavior of N proteins between the different strains of HP-PRRSV (JXA1 and JXA1-R) and classical PRRSV (VR2332).

### 3.3 Differential expression of N protein mRNA and nuclear translocation receptor mRNA during infection of the three strains of the virus

**Figure 3:**
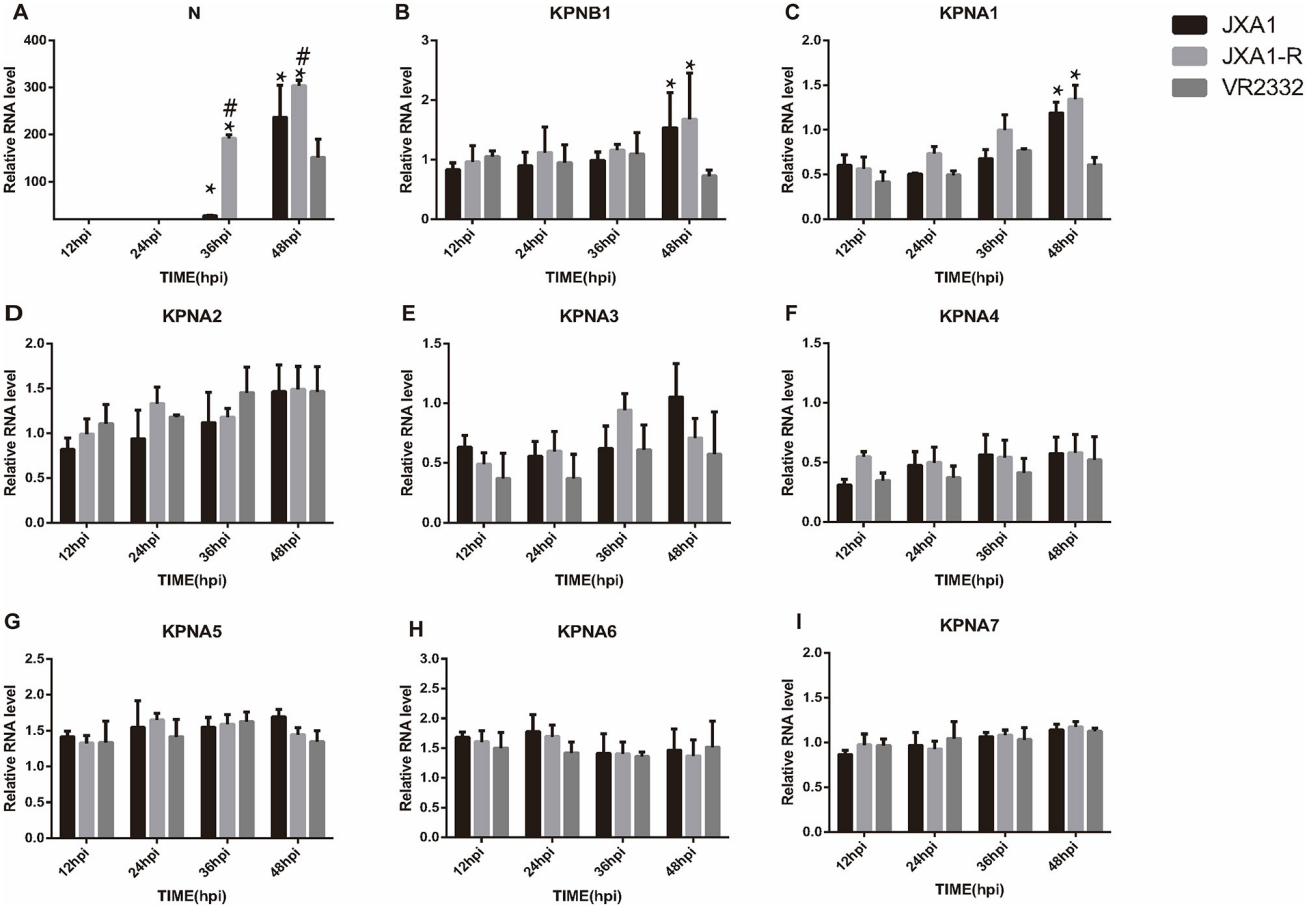
Analysis of mRNA expression differences of related proteins among three strains at the same infection time. “*” indicates that JXA1-R and JXA1 were compared with VR2332, respectively, *p* < 0.01; “#” indicates that JXA1-R was compared with JXA1, *p* < 0.01. No annotation indicates no statistically significant difference.

Quantitative real-time PCR analysis was conducted on PRRSV strains cultured in Marc-145 cells at a MOI = 0.1 at different times. The expression trends of N protein and nuclear translocation receptor mRNA expression were observed in different strains at the same culture length and in the same strain at different culture lengths. We found that JXA1, JXA1-R, and VR2332 showed that the N protein mRNA increased with the increase in incubation time and a sudden increase was observed at 36 hpi, 36 hpi, and 32 hpi, respectively. In addition, the expression levels of KPNB1, KPNA1, KPNA2, KPNA4, KPNA6, and KPNA7 changed with the infection time of the three strains while KPNA3 and KPNA5 did not change with the incubation time (Supplementary Figures S4–S6). The expression levels of N protein mRNA of the three strains differed at the same infection time, with the order being JXA1-R>JXA1>VR2332 (Figure 3A). The expression trends of KPNB1 and KPNA1 were VR2332 < JXA1 and VR2332 < JXA1-R and the differences between JXA1 and JXA1-R were not statistically significant (Figures 3B, C). As depicted, the replication efficiency of classical PRRSV was lower than that of HP-PRRSV, with the expression of nuclear translocation receptors KPNB1 and KPNA1 being different between classical PRRSV and HP-PRRSV infected with Marc-145 cells.

### 3.4 Knockdown of KPNA1 reduces the efficiency of HP-PRRSV replication

**Figure 4:**
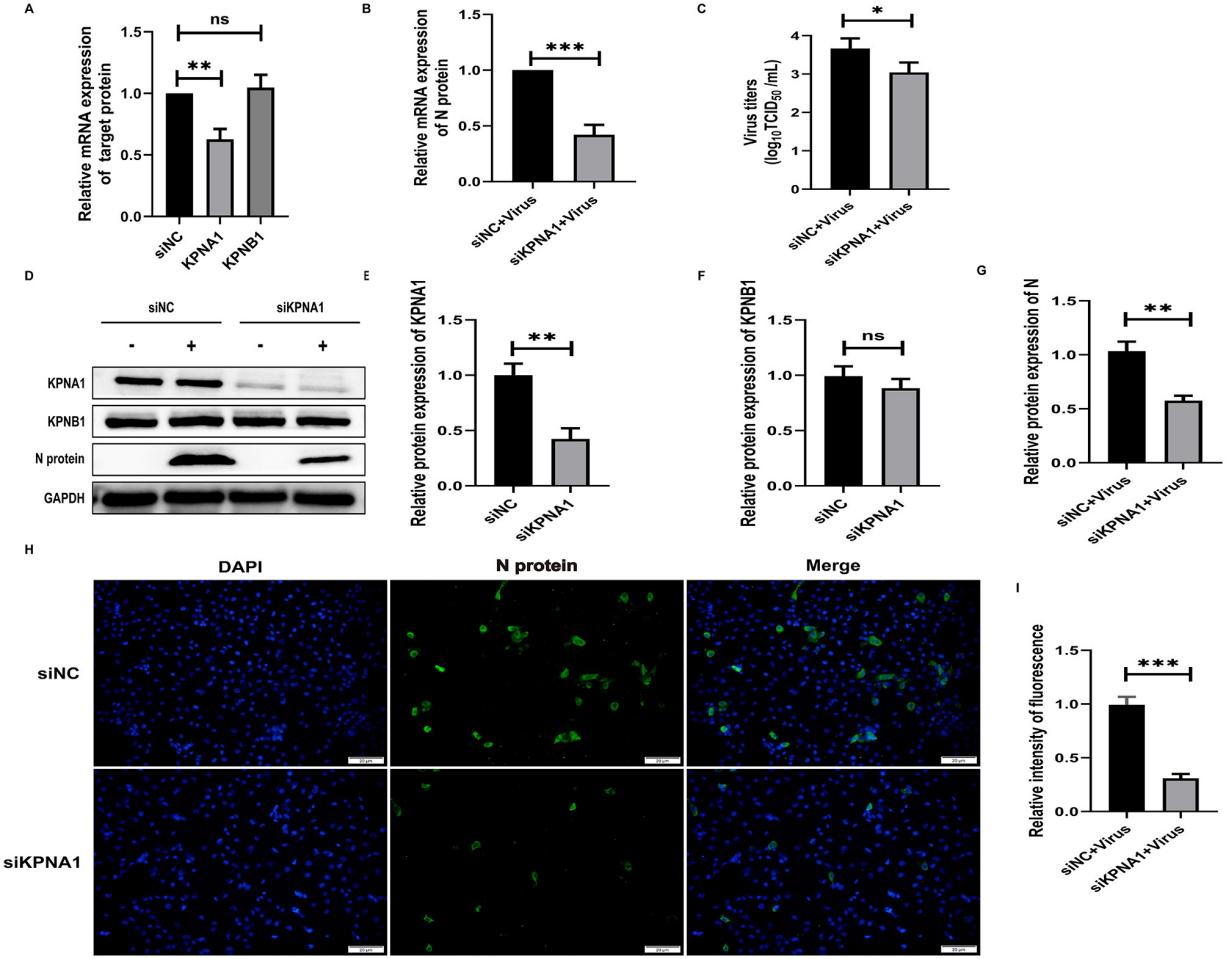
Knockdown of KPNA1 inhibits JXA1 replication efficiency. Marc-145 cells were transfected with siKPNA1 for 48 h, followed by infection with JXA1 at MOI = 0.1 for 24 h. **(A,B)** qPCR was used to detect the knockdown efficiency of KPNA1 and the mRNA level of JXA1 N protein. **(C)** TCID50 was used to detect the viral titer in cell supernatant. **(D–G)** Western blot assay was used to detect the expression levels of related proteins. **(H,I)** Cell immunofluorescence assay was used to monitor JXA1 N protein replication. Image J software was used to analyze the gray values and fluorescence intensity of the protein bands. *p < 0.05, **p < 0.01, ***p < 0.001, ns indicates no statistical significance.

To investigate the role of KPNA1 in the nuclear transport of the PRRSV N protein and the quantitative relationship between KPNA1 and KPNB1, we first treated the cells with siRNA to reduce the expression levels of KPNA1 in Marc-145 cells for 48 h and monitored the relevant indexes using qPCR, virus titration, western blot assay, and cell immunofluorescence assay after incubation with the cells for 24 h. The qPCR results showed that the KPNA1 expression was significantly down-regulated, compared with the siNC group (*p* < 0.001). The changes of KPNB1 were statistically insignificant (*p* > 0.05; Figure 4A), with a notable reduction in the JXA1 N protein (*p* < 0.001; Figure 4B). Virus TCID_50_ titration showed a statistically significant (*p* < 0.05) reduction in the viral titer of HP-PRRSV in the siKPNA1+Virus group compared to the siNC+Virus group (Figure 4C). Moreover, western blot assay showed that compared with siNC group, the change of KPNB1 was statistically insignificant (*p* > 0.05) when the expression of KPNA1 was down-regulated (*p* < 0.01) while the decrease of JXA1 N protein was statistically significant (*p* < 0.01; Figures 4D–G). Cell immunofluorescence assay showed a lower amount of green fluorescence of N protein in the siKPNA1+Virus group compared to the siNC+Virus group and the statistical analysis indicated a decrease in the replication efficiency of HP-PRRSV (JXA1 ) (*p* < 0.01; Figures 4H, I).

### 3.5 Knockdown of KPNB1 reduces HP-PRRSV replication efficiency

**Figure 5:**
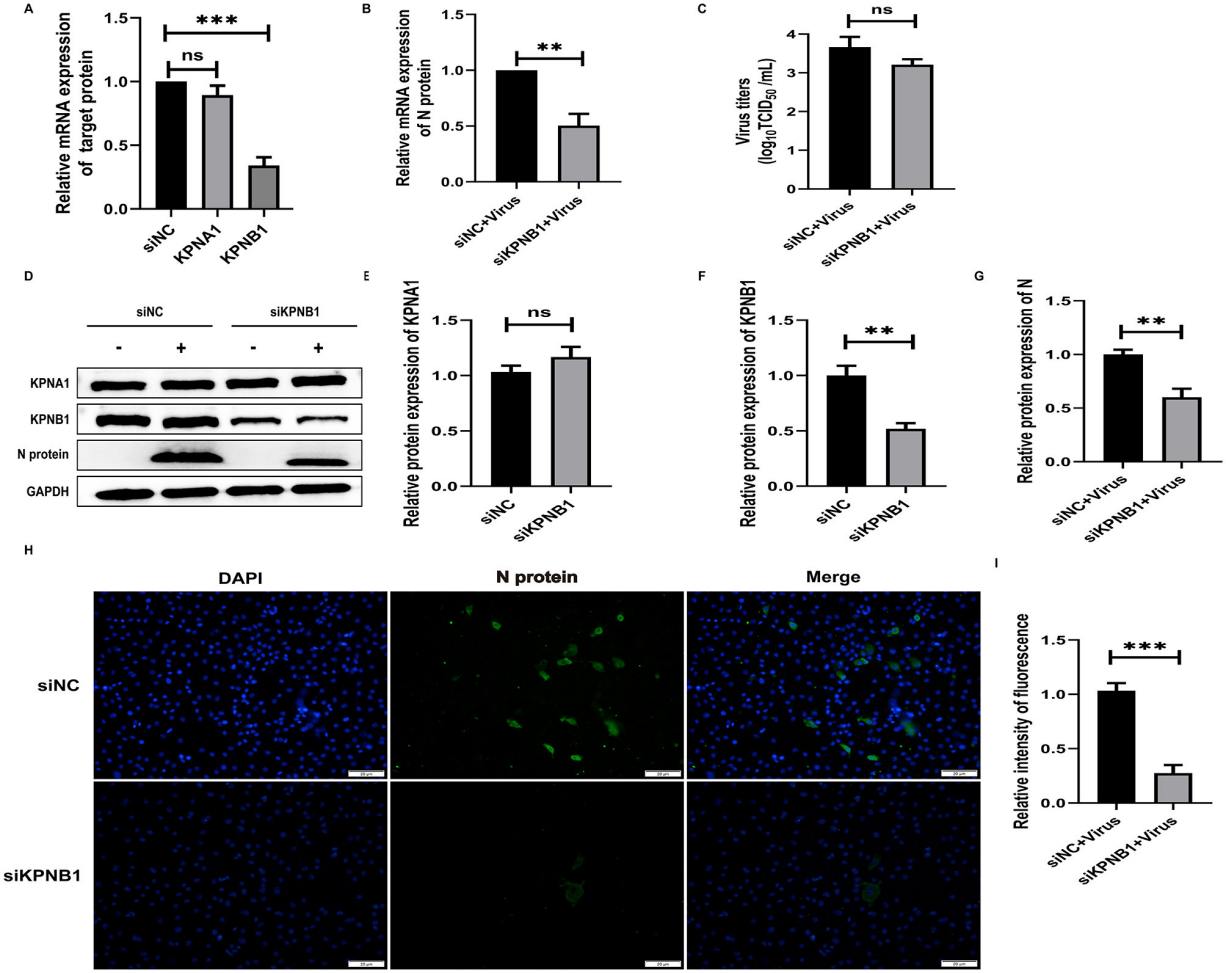
Knockdown of KPNB1 inhibits JXA1 replication efficiency. Marc-145 cells were transfected with siKPNB1 for 48 h, followed by infection with JXA1 at MOI = 0.1 for 24 h. **(A,B)** qPCR was used to detect the efficiency of KPNB1 knockdown and the mRNA level of JXA1 N protein. **(C)** TCID50 was used to detect the viral titer in cell supernatant. **(D–G)** Western blot assay was used to detect the expression levels of related proteins. **(H,I)** Cell immunofluorescence assay was used to monitor JXA1 N protein replication. Image J software was used to analyze the gray values and fluorescence intensity of the protein bands. **p < 0.01, ***p < 0.001, ns indicates no statistical significance.

To investigate the role of KPNB1 in the nuclear transport of JXA1 N protein and the quantitative relationship between KPNB1 and KPNA1, we first used siRNA treatment for 48 h to reduce the expression level of KPNB1 in Marc-145 cells and then monitored the relevant indexes by qPCR, virus titration, western blot, and cell immunofluorescence assays after 24 h incubation with the virus. The qPCR results showed that the expression levels of KPNB1 were significantly down-regulated (*p* < 0.001) compared with that of the siNC group. The qPCR results showed that the expression level of KPNB1 was significantly down-regulated (*p* < 0.001) compared with that of the siNC group. However, the change in KPNA1 was not statistically significant (*p* > 0.05; Figure 5A). N protein exhibited statistically significant results (*P* < 0.001; Figure 5B). Moreover, the virus TCID_50_ Titration showed that the viral titer of JXA1 in siKPNB1+Virus group was reduced but not statistically significant (*p* > 0.05) compared with the siNC+Virus group (Figure 5C). Western blot assay showed that compared with the siNC group, the change of KPNA1 was not statistically significant (*p* > 0.05) when the expression of KPNB1 was down-regulated (*p* < 0.01) and a decrease of JXA1 N protein was statistically significant (*p* < 0.01; Figures 5D–G). The results showed that the number of green fluorescence of the N protein was less in the siKPNB1+Virus group compared with the siNC+Virus group and the statistical analysis indicated that the replication efficiency of HP-PRRSV (JXA1) was reduced (*p* < 0.01; Figures 5H, I).

### 3.6 Simultaneous knockdown of KPNA1 and KPNB1 reduces HP-PRRSV replication efficiency

**Figure 6:**
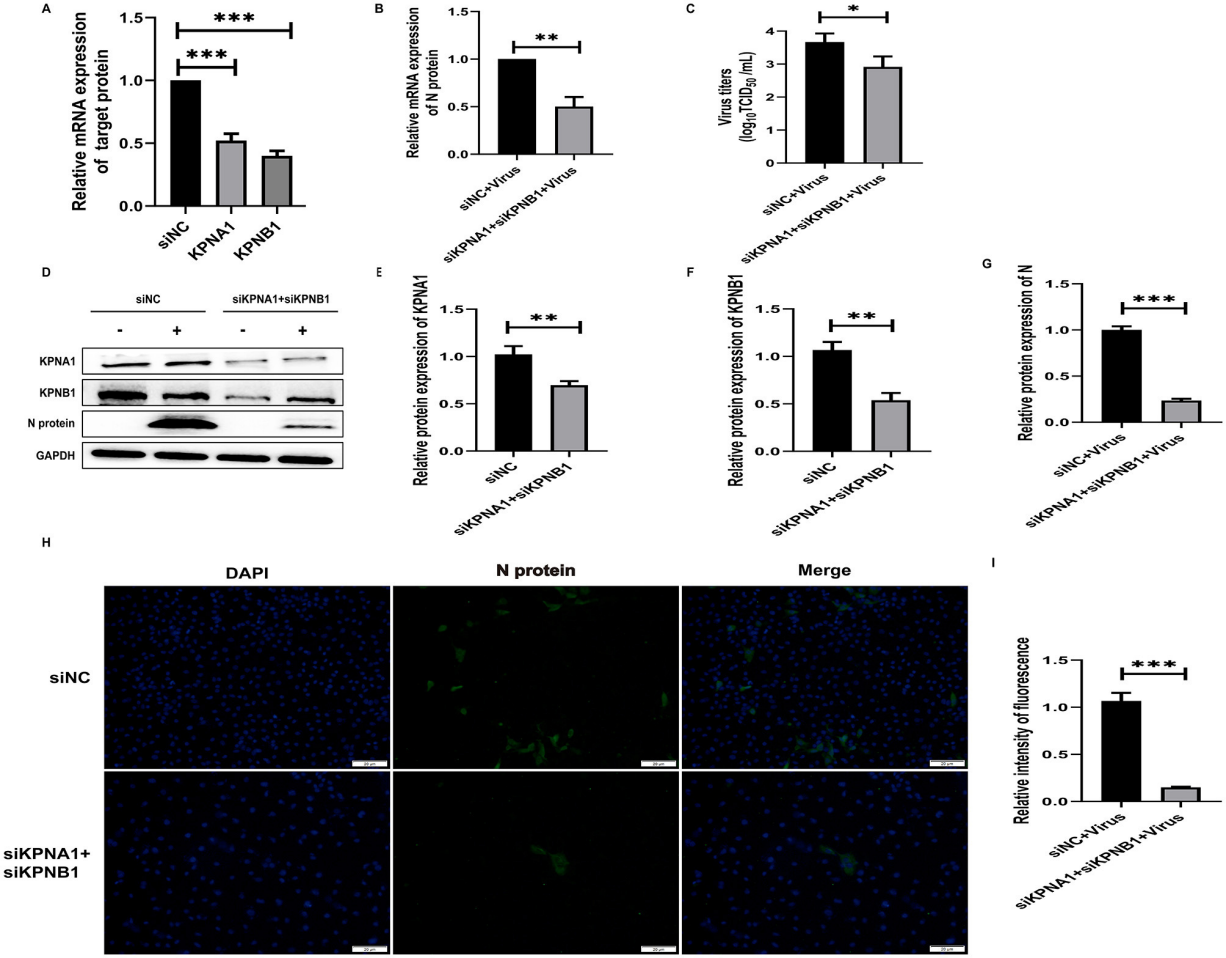
Simultaneous knockdown of KPNA1 and KPNB1 inhibits JXA1 replication efficiency. After co-transfection of Marc-145 cells with siKPNA1 and siKPNB1 for 48 h, JXA1 was added at MOI = 0.1 for 24 h. **(A,B)** qPCR was used to detect the knockdown efficiency of KPNA1 and KPNB1 and the mRNA level of JXA1 N protein. **(C)** TCID50 was used to detect the viral titer in the cell supernatant. **(D–G)** Western blot assay was used to detect the expression levels of related proteins. **(H,I)** Cell immunofluorescence assay was used to monitor JXA1 N protein replication. Image J software was used to analyze the gray scale values and fluorescence intensity of protein bands. *p < 0.05, **p < 0.01, ***p < 0.001.

To investigate the joint role of KPNA1 and KPNB1 in the nuclear transport of PRRSV N protein, we reduced the expression levels of KPNA1 and KPNB1 in Marc-145 cells by siRNA treatment for 48 h and then monitored the relevant indexes by qPCR, virus titration, western blot assay, and cell immunofluorescence assay after 24 h of virus incubation. The qPCR and western blot assay results showed that the expression levels of KPNA1 and KPNB1 were comparable with those of the siNC group. Then, qPCR, Virus titration, western blot assay, and cell immunofluorescence assay were used to monitor relevant indexes after 24 h of virus incubation. In addition, qPCR and western blot assay showed that the expression levels of KPNA1 and KPNB1 were significantly down-regulated compared to that in the siNC group (*p* < 0.001; Figures 6A, D–F) and the expression level of JXA1 N protein was also significantly down-regulated in Marc-145 cells (*p* < 0.001; Figure 6A). JXA1 N protein was also decreased (*p* < 0.001; Figures 6B, G). Virus TCID_50_ titration showed that the viral titer of JXA1 in siKPNA1+siKPNB1+Virus group was decreased compared with siNC+Virus group (*p* < 0.05; Figure 6C). Cell immunofluorescence assay showed less green fluorescence of N protein in siKPNA1+siKPNB1+Virus group compared with siNC+Virus group, and the statistical analysis showed that the replication efficiency of HP-PRRSV (JXA1) was reduced (*p* < 0.01; Figures 6H, I).

### 3.7 Protein binding model prediction and molecular dynamics analysis

**Figure 7:**
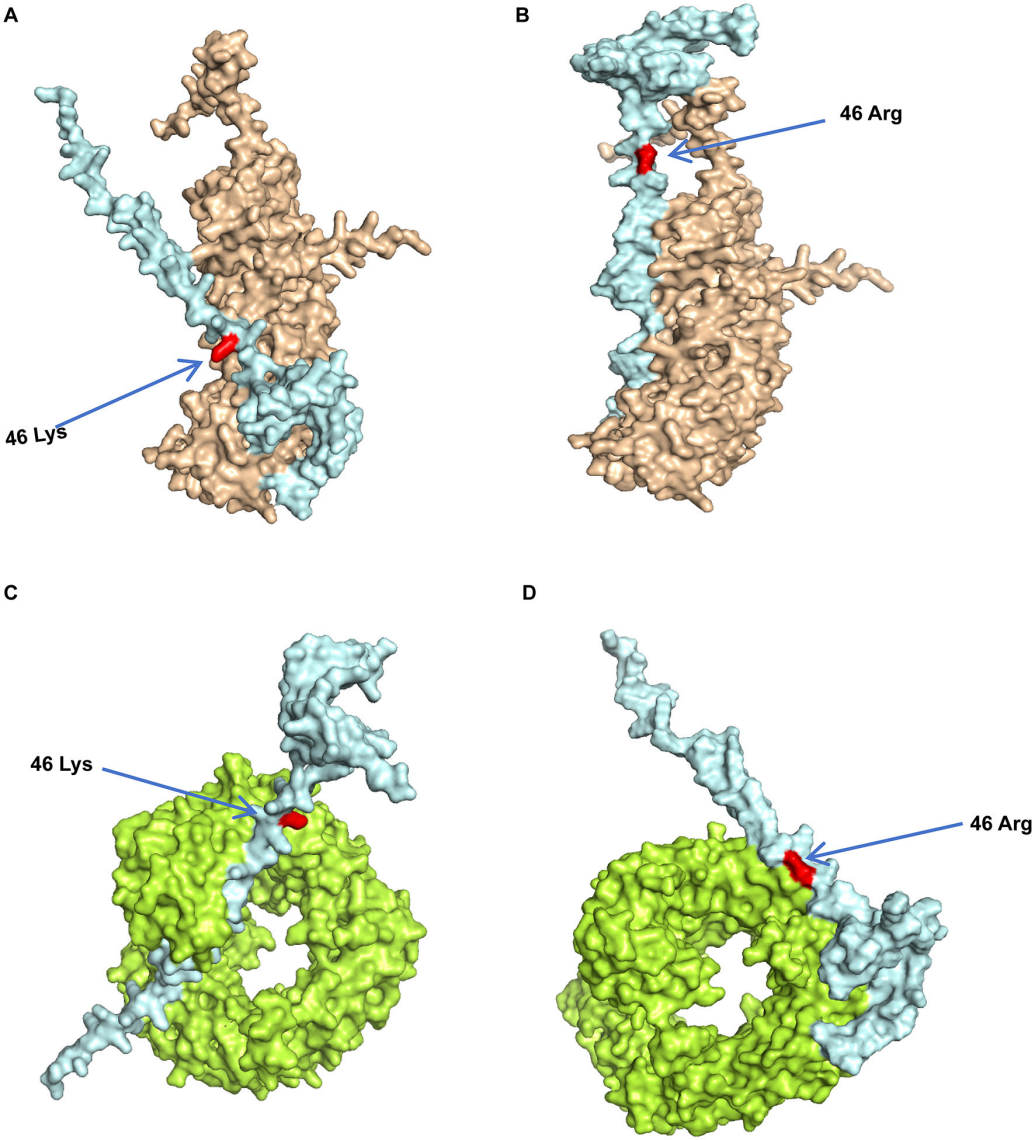
Predicted model of PRRSV N protein binding to receptor proteins. The three-dimensional structures of the proteins were obtained from UniPort and Alphafold3. **(A)** Model of Classic PRRSV N protein binding to KPNA1, where light blue represents N protein, brown represents KPNA1, and red represents 46K. **(B)** Model of HP-PRRSV N protein binding to KPNA1, where light blue represents the N protein, brown represents KPNA1, and the red site is 46R. **(C)** Classic PRRSV N protein binding model with KPNB1, where light blue represents the N protein, green represents KPNB1, and the red site is 46K. **(D)** HP-PRRSV N protein binding model with KPNB1, where light blue represents the N protein, green represents KPNB1, and the red site is 46R.

**Figure 8:**
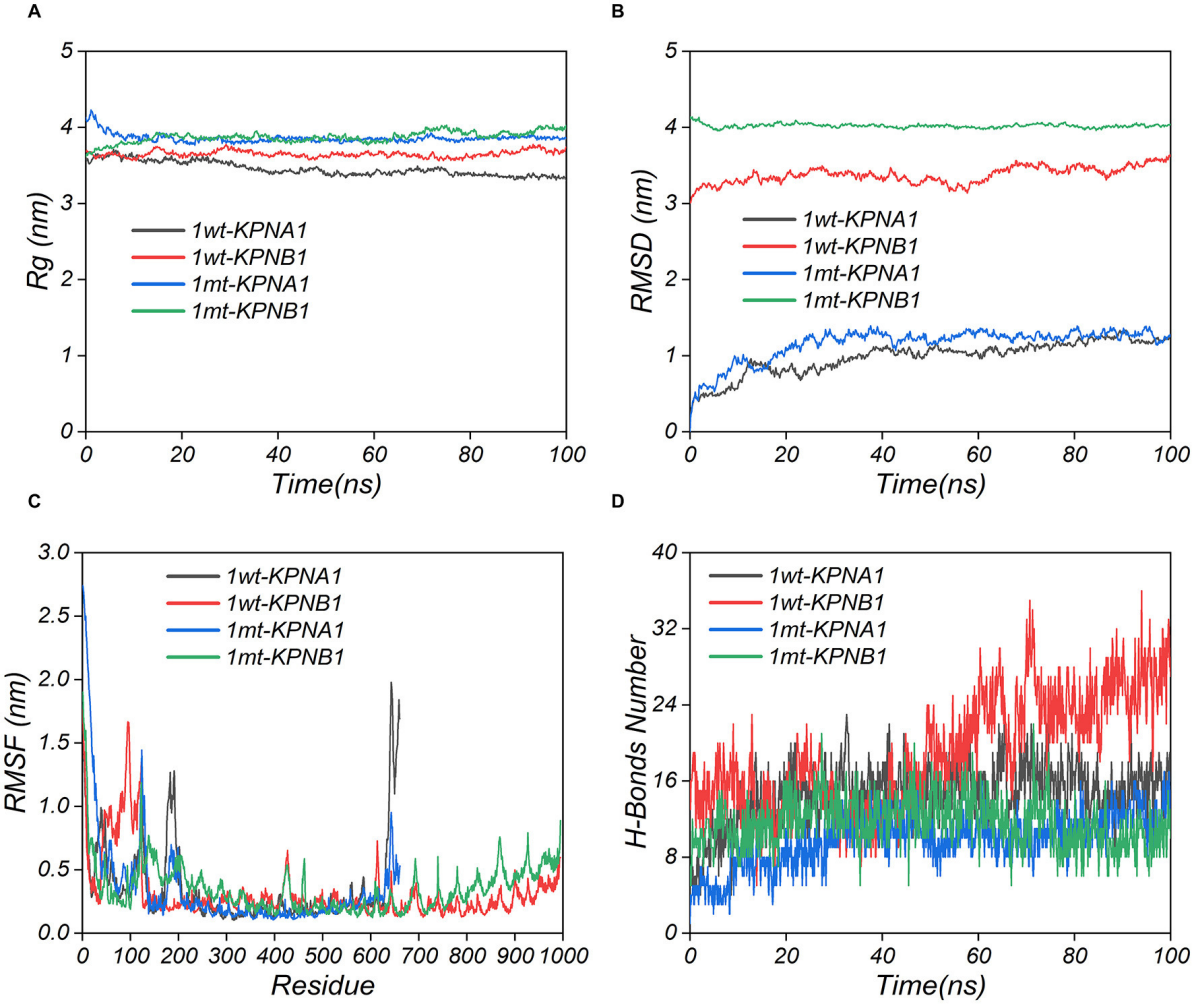
Kinetic simulation analysis of PRRSV N protein binding to receptor proteins. The three-dimensional structures of the proteins were obtained using UniPort and Alphafold3. wt refers to Classic PRRSV N protein, and mt refers to HP-PRRSV N protein.

The modeled structures of the classic PRRSV N protein and HP-PRRSV N protein bound to KPNA1 and KPNB1 showed a change in the binding conformation (Figure 7). In the 100 ns simulation, the Rg values of the complexes fluctuated in the first and middle stages of the simulation due to the equilibrium of the system and then remained stable in the late stage of the simulation. The wt-KPNA1 (classic PRRSV N protein-KPNA1) values were maintained around 3.3 nm, the wt-KPNB1 (classic PRRSV N protein-KPNB1) values around 3.8 nm, and mt-KPNB1 (HP-PRRSV N Protein-KPNB10) and mt-KPNA1 (HP-PRRSV N protein-KPNA1) values around 4.0 nm. Notably, the proteins in the wt group maintain a more compact structure while proteins in the mt group exhibit higher Rg values. Since the KPNB1 protein is larger compared to the KPNA1 protein, it has a correspondingly higher Rg value (Figure 8A). Similarly, the RMSD values of the proteins fluctuated in the early phase of the simulation due to equilibrium and the fluctuations decreased. The trend of the RMSD data was like that of the Rg data. Generally, the KPNA1-bound proteins had lower RMSD values for both wt and mt while the KPNB1 group had higher RMSD values. Moreover, the KPNB1 group had reached equilibrium at the beginning of the generative phase of the simulation (Figures 8A, B). For the two systems of the wt protein, the main structural fluctuations mainly appeared around a residue 100, except for the fluctuations of N protein and the C-terminal of wt protein. After binding to wt protein, fluctuations of KPNA1 and KPNB1 proteins were relatively small (RMSF < 1 nm); no large fluctuations were found except for the two ends of the proteins. In the context of the mt protein, the fluctuation region spanned a larger area, mainly the residues near the C-terminus of the protein. Similarly, KPNA1 and KPNB1 proteins did not show large fluctuations upon binding to mt proteins (Figure 8C). We analyzed the information on hydrogen bond formation between N protein-receptor proteins. Then, 100 ns molecular dynamics simulations showed that hydrogen bond interactions could be formed between N protein-receptor proteins and the average number of hydrogen bonds for each system was 14.57 (wt-KPNA1), 19.07 (wt-KPNB1), 9.79 (mt-KPNA1), 11.56 (mt-KPNA1), 11.56 (mt-KPNA1), 11.56 (mt-KPNA1), and 11.56 (mt-KPNA1), respectively. Moreover, by further analyzing the binding free energies of the four pairs of the systems, 11.56 (mt-KPNB1) (Figure 8D), we obtained the values of binding free energies for the corresponding systems as −154.61 ± 15.14 kcal/mol (wt-KPNA1), −165.38 ± 24.54 kcal/mol (wt-KPNB1), −146.69 ± 18.79 kcal/mol (mt-KPNA1), and −145.57 ± 12.09 kcal/mol (mt-KPNB1). Since negative energy values indicate binding and larger absolute values indicate stronger binding, it was evident that the binding strength of HP-PRRSV N protein to its receptor proteins, KPNA1 and KPNB1, was reduced compared with that of classical PRRSV.

### 3.8 Protein binding bio-layer interferometry detection and analysis

**Figure 9:**
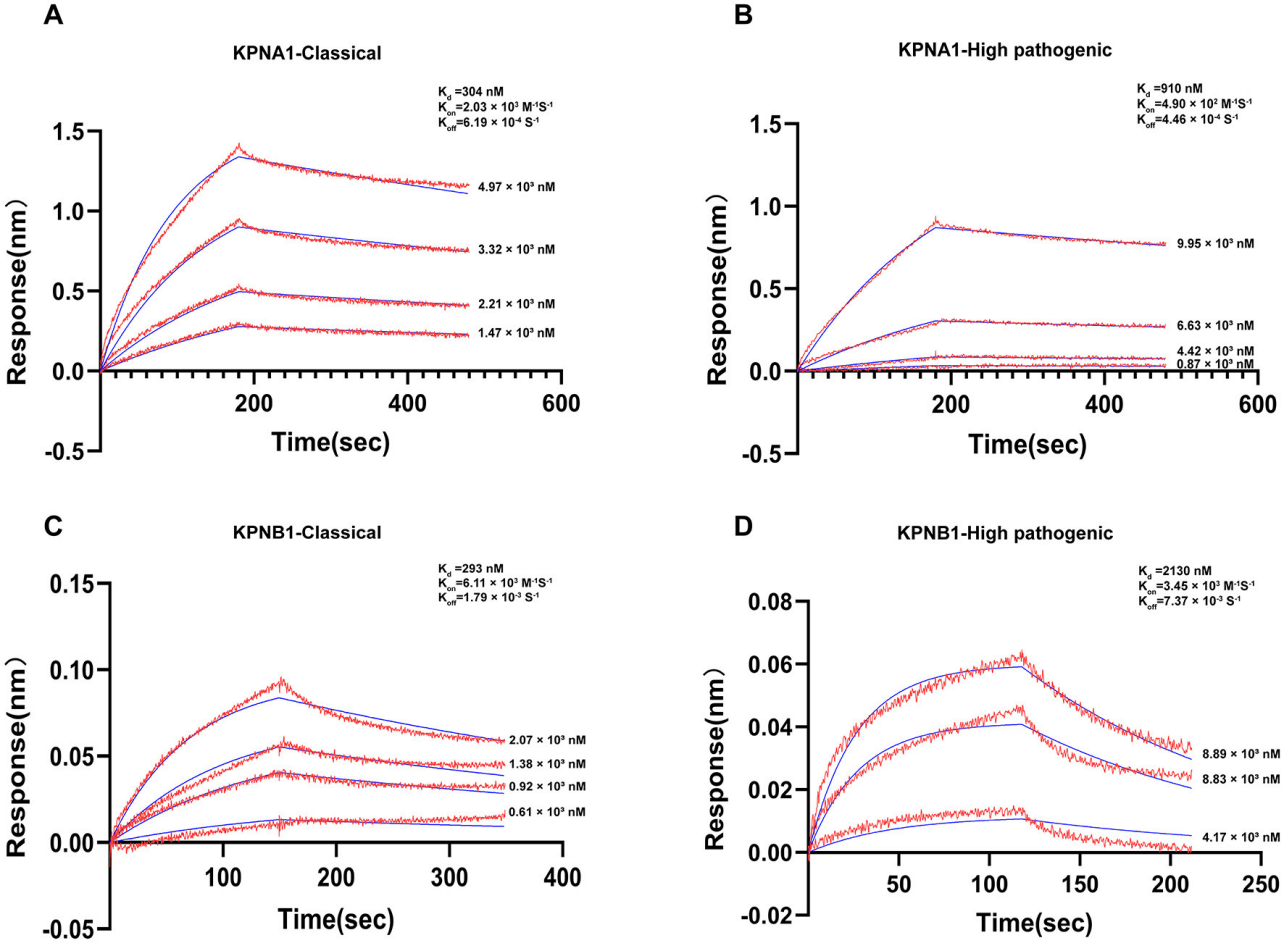
Actual binding parameters of PRRSV N protein and receptor protein. The detection temperature was 30°C, and the buffer was PBS + 0.2% BSA. Red indicates the actual binding curve, blue indicates the fitted curve, and the Kd value was calculated using a 1:1 binding model. **(A)** SA probe immobilized Classic PRRSV N protein, binding concentration was Classic PRRSV N protein (10 μg/ml, immobilization height 2.2 nm) + KPNA1 (gradient concentration 1.47–4.97 μM). **(B)** SA probe immobilized HP-PRRSV N protein, binding concentration was HP-PRRSV N protein (10 μg/ml, immobilization height 2.2 nm) + KPNA1 (gradient concentration 0.87–9.95 μM). **(C)** APS probe immobilized KPNB1, binding concentration of KPNB1 (10 μg/ml, immobilized to saturation) + Classic PRRSV N protein (gradient concentration of 0.61–2.07 μM). **(D)** APS probe solidified KPNB1, binding concentration of KPNB1 (10 μg/mL, solidified to saturation) + HP-PRRSV N protein (gradient concentration of 4.17–8.89 μM).

The constants for equilibrium dissociation (K_d_), the association rate (K_on_), and the dissociation rate (K_off_) were identified to determine the binding of classic PRRSV and HP-PRRSV N protein to KPNA1 and KPNB1. BLI analyses were conducted using ForteBio's Octet optical biosensor system (Sartorius, Fremont, CA, United States). The results showed that both classic PRRSV and HP-PRRSV N proteins could effectively bind to KPNA1 with K_d_ values of 304 nM (K_on_ = 2.03 × 10^3^ M^−1^ S^−1^, K_off_ = 6.19 × 10^−4^ S^−1^) and 910 nM (K_on_ = 4.90 × 10^2^ M^−1^ S^−1^, K_off_ = 4.46 × 10^−4^ S^−1^) based on kinetic data. This shows that the classic PRRSV N protein binds more strongly to KPNA1 than HP-PRRSV (Figures 9A, B). Similarly, classic PRRSV and HP-PRRSV N proteins could also bind KPNB1 efficiently with K_d_ values of 293 nM (K_on_ = 6.11 × 10^3^ M^−1^ S^−1^, K_off_ = 1.79 × 10^−3^ S^−1^) and 2,130 nM (K_on_ = 3.45 × 10^3^ M^−1^ S^−1^, K_off_ = 7.37 × 10^−3^ S^−1^), respectively. The kinetic data showed that the classic PRRSV N protein binds KPNB1 much more strongly than the HP-PRRSV (Figures 9C, D).

### 3.9 Summary of evidence for mutations in key amino acids (K and R)

To further investigate whether the identified mutations in the key NLS amino acids (K → R or R → K) are representative, we combed through the literature and concluded that such mutations are universal as shown in Table 2.

**Table 2:** Evidence related to mutations in the key amino acids (K and R).

**Article type**	**Amino acid mutation status**	**Post-mutation effects**	**References**
Research article	IRF3 is located in the NLS region with a K77R mutation.	Significantly restored the degradation of IRF3 and its nuclear translocation without affecting the NLS activity of IRF3.	Liu et al., 2024
Review article	K356R mutation in the spiking receptor binding domain of SARS-CoV-2 viruses of animal origin.	Reduces the binding of human antiserum to spiny proteins by 10–20% and is located on the surface of the RBD, overlapping with antigenic epitope 3.	Zhao et al., 2024
Research article	K101R mutation in Zika virus (ZIKV) capsid (C) protein.	Enhanced viral neurotoxicity by making the virus replicate more efficiently in the neonatal mouse brain and inducing a stronger inflammatory response.	Song et al., 2023
Research article	K247R and R247K mutations in The Newcastle disease virus (NDV) matrix (M) protein located in the mixed NLS and nuclear export signal (NES) region (247–266).	Enhances viral replication and virulence and drives nucleoplasmic transport.	Peng et al., 2022
Research article	K214R mutation in the NLS2 region of the influenza A virus nucleoprotein (NP).	Attenuates the binding of NLS2 to importinα, which in turn leads to reduced nuclear import capacity.	Wu et al., 2017
Research article	Avian influenza H9N2 virus PA protein K356R mutation	Increased accumulation of viral PA nuclei and enhanced viral polymerase activity in human A549 cells, which in turn led to elevated levels of viral transcription and viral export, also enhanced viral replication and caused lethal infection in mice.	Xu et al., 2016

## 4 Discussion

In the field of viral infection research, NLS has garnered significant attention due to its vital role in viral nucleoplasmic shuttling, replication, and pathogenicity. Numerous studies have shown that NLS essentially impacts the infection processes of most viruses. For example, the NLS of the influenza virus NP plays a critical role in regulating the nuclear import of the virus into the host cell, and mutations in its NLS can severely disrupt the viral replication cycle and impede the normal proliferation of the virus (Wu et al., 2017). The NDV has also shown that mutations in the NLS of the matrix (M) protein can alter the efficiency of nucleoplasmic shuttling for this protein. These changes subsequently affect the assembly and release of the virus, ultimately leading to alterations in the pathogenicity of the virus (Peng et al., 2022). Furthermore, the capsid protein of the Venezuelan equine encephalitis virus facilitates infection by inhibiting the synthesis of β-interferon in the host cell after nucleation. Moreover, the virulence of the virus is substantially reduced when the NLS sequence of the nucleation protein is mutated (Atasheva et al., 2010). Dengue virus non-structural protein 5 can resist the antiviral effects of the host cell after nucleation, including the inhibition of IL-8 synthesis in the target cells. When the NLS sequence of NS5 protein is mutated, the viral replication capacity is reduced approximately by 1,000-fold (Pryor et al., 2007). These studies highlight the centrality of NLS in the mechanism of viral infection. However, previous studies on PRRSV have mainly focused on the analysis of the viral genome sequence, immune response, and the preliminary investigation of the function of some proteins. The role of NLS in the nucleoplasmic shuttling of viruses and their pathogenicity has been explored in only a few studies (Su et al., 2024; Luo et al., 2020; Ke et al., 2019), indicating a lack of in-depth comprehension of these phenomena. In this study, we uniquely explored the mechanisms by which HP-PRRSV enhances pathogenicity from the perspective of nucleoplasmic shuttling, thereby filling the existing gap in this field. Through rigorous experiments and in-depth analyses, we found and confirmed that mutations in key amino acids in the NLS have significantly influenced the nucleoplasmic shuttling of the virus. Furthermore, we revealed the underlying mechanism by which this effect is closely related to receptor binding. In addition, we also predicted that the mutations of key amino acids (K → R or R → K) in the NLS region might be universally applicable to the binding of viral nucleoprotein to its receptor (Table 2), providing a valuable reference and a new research direction for the future related studies.

The structure and chemistry of amino acids play a decisive role in the functionality of proteins. To explore the mechanism by which mutations in single amino acids affect the binding of nucleolar proteins to their receptors, thorough analyses of the molecular levels are required. Both lysine and arginine are classified as basic amino acids; however, their side-chain structures are markedly different; the side-chain of K contains a primary amino group whereas the side-chain of R contains a guanidino group. These structural differences affect their ability to form various bond energies, bond angles, and van der Waals forces with surrounding amino acid residues. In the present study, the mutation of lysine at position 46 within the NLS region of the PRRSV N protein to arginine has seemingly resulted in a modification in the local spatial conformation (Figure 7). Moreover, the binding affinity in protein interactions is primarily influenced by various forces. When lysine is mutated to arginine, it triggers a series of changes at the chemical energy level, substantially decreasing the binding affinity between proteins. In addition, lysine and arginine had different pKa values, leading to different ionization behaviors under physiological pH conditions. The primary amine of the lysine side chain group, which is positively charged, can form an electrostatic attraction with the negatively charged regions of the receptor protein. This interaction is vital for maintaining interprotein binding, however, after mutation to arginine, the charge distribution of its guanidinium group was changed. While the arginine was also positively charged, the pattern of its electrostatic interaction with the receptor differed, weakening the stable electrostatic attraction initially formed by lysine, consequently leading to decreased binding affinity. Secondly, there is also a change in hydrogen bonding. The primary amine group of lysine can act as a hydrogen bond donor to form hydrogen bonds with acceptor proteins, stabilizing the interprotein binding conformation. However, the guanidinium group structure of arginine made its hydrogen bonding ability different from that of lysine; after the mutation, the number and strength of hydrogen bonds formed with receptor proteins in the original position were altered. The results of this study showed that the average number of hydrogen bonds between the classic PRRSV N protein and the receptor protein was reduced after mutation. Specifically, when the classical PRRSV was bound to KPNA1, the number of hydrogen bonds was 14.57 which was reduced to 9.79 for the mutated HP-PRRSV. Similarly, when classical PRRSV was bound to KPNB1, the number of hydrogen bonds was 19.07, reduced to 11.56 for the mutated HP-PRRSV. In addition, van der Waals forces were also altered by the amino acid mutations. These forces include the dispersive, induced, and orientational, with their magnitude and direction being contingent on the shape of the molecule, distribution of the electron cloud, and relative position. When lysine is mutated to arginine, the change inside the chain structure triggers a local spatial conformational change, thereby affecting the spatial fitness between the protein and the receptor and causing deviations from the originally fitted region. Consequently, this induces a redistribution of van der Waals' forces between the surrounding amino acid residues. The new spatial conformation leads to unfavorable combinations of van der Waals forces for binding, ultimately reducing the binding affinity. In addition, changes in spatial conformation, commonly caused by amino acid mutations, have significantly impacted inter-protein binding in various studies related to viral proteins. This further illustrates the role of this mechanism in decreasing the binding affinity associated with the mutation of lysine to arginine (Li et al., 2022; Nacken et al., 2021; Deng et al., 2021). The combined effects of the above decrease the binding strength of the HP-PRRSV N protein to its receptor proteins KPNA1 and KPNB1. This reduction subsequently affects the efficiency of the nucleoplasmic shuttling of the functional viral N protein.

In this study, we observed that the mutated HP-PRRSV N proteins in the NLS region have a reduced binding affinity to their receptor proteins, KPNA1 and KPNB1. However, this alteration increased the efficiency of the N proteins entering the nucleus, leading to a substantial, more efficient replication of HP-PRRSV than classic PRRSV. We hypothesize that the following factors and mechanisms may be at play: (1) Nuclear transport receptors increased kinetic rate and transport capacity. Previous researchers stated that lower binding affinity indicates faster dissociation, indicating that nuclear transporter receptors of KPNA1 and KPNB1 dissociate more rapidly from N proteins, enhancing their ability to re-engage in new transport cycles (Fradin et al., 2005; Görlich and Kutay, 1999); (2) Conformational changes of N proteins including mutations in the NLS region which alter the conformation of N proteins, causing them to interact with those in N proteins (Figure 7). This results in altered interactions with nuclear translocation receptors and nuclear pore complexes, ultimately facilitating the entry of N proteins into the host cell nucleus through the nuclear pore complexes similar to the findings of previous research (Ibáñez de Opakua et al., 2024). During viral replication, the inability of viral proteins to effectively enter the nucleus hinders their capacity to execute essential functions, such as replication and transcription of the viral genome. In addition, viral proteins that fail to enter the nucleus properly will be unable to interact with other viral proteins, preventing the formation of complete viral particles. Therefore, accurate localization of viral proteins within the nucleus is a crucial step in the viral life cycle. The functional N proteins of the hp-PRRSV exhibit efficient nucleation, consequently, increasing the efficiency of their replication; and (3) the N proteins exhibiting mutations in the key amino acids of the NLS may reduce the antiviral response. Research has shown that the N protein of PRRSV can activate the NF-κB signaling pathway (Su et al., 2024). NF-κB is a class of nuclear transcription factors exhibiting various roles. When activated, it participates in transcriptional regulation of most genes and plays an important role in immunity, inflammation, oxidative stress, cell proliferation, apoptosis, and other physiopathological processes (Ito et al., 2025; Hou et al., 2025; Zeng et al., 2024). When the key amino acids of the NLS are mutated, the HP-PRRSV N protein may interact less with the immune-related factors in the nucleus, thus maximizing its ability to evade the immune attack of the host cells. These factors may also reflect the adaptive strategies employed in the evolution of HP-PRRSV. This study holds significance for the in-depth understanding of the pathogenic mechanism of PRRSV. It has the key roles of the key amino acid mutations in the NLS region that differentiate HP-PRRSV from classical PRRSV, providing molecular insights for future investigations into the evolutionary pathway of the virus. Meanwhile, the analyses of the binding mechanism between the N protein and nuclear transporter receptor can facilitate further understanding of how viruses utilize the nuclear transport system of host cells for replication and propagation. This study also identified potential targets for developing new antiviral drugs aimed at disrupting the viral nucleoplasmic shuttling process. For example, future efforts could focus on designing specific small molecule inhibitors that impede the binding of the nuclear translocation receptor to mutant N proteins. Furthermore, the results of this study can provide theoretical insights that can facilitate the development of strategies for PRRSV prevention and control. In the context of vaccine development, the design of key amino acid mutation sites in the NLS region can be considered to enhance the vaccine-induced immune response and improve the prevention and control of PRRSV. It can provide timely warnings regarding the emergence of highly pathogenic strains by detecting mutations in the key amino acids in the NLS region, enabling the implementation of appropriate preventive and control measures to mitigate economic losses and promote the sustainable growth of the pig breeding industry. Meanwhile, this study still has some limitations. Firstly, although the effect of the natural mutation of the 46th key amino acid (K46R) in the NLS region on the nucleoplasmic shuttling and replication efficiency of HP-PRRSV has been confirmed through protein binding simulation, kinetic analysis, and cellular experiments, functional verification based on reverse genetics technology has not yet been carried out—reverse transcription mutant strains that restore the K46R site in classic strains to wild-type K have not yet been constructed and functionally characterized. Our laboratory is committed to establishing a reverse genetics technology platform. By constructing HP-PRRSV reverse transcription mutant strains and conducting functional studies, we aim to clarify the causal relationship of K46R mutation in viral pathogenesis. Secondly, the present study only revealed the patterns of viral replication and nucleoplasmic shuttling at the Marc-145 cell level, lacking evidence of pathogenicity *in vivo*. To explore the influence of the N protein mutation of HP-PRRSV on the pathogenicity of the virus, porcine infection experiments will be carried out subsequently. By comparing the pathogenicity differences of wild-type, mutant and reverse mutant strains, and combining immunological indicators to analyze the influence of the mutation on the virus-host interaction, a more comprehensive experimental basis will be provided for clarifying the pathogenic mechanism of HP-PRRSV.

## Data Availability

The raw data supporting the conclusions of this article will be made available by the authors, without undue reservation.
