# Supplementary figures and images for: Natural mutations in key NLS amino acids regulate nucleoplasmic shuttling and replication efficiency in PRRSV
Source: Front Microbiol. 2025 Jul 4;16:1587634. doi: 10.3389/fmicb.2025.1587634 (PMC12271210; doi:10.3389/fmicb.2025.1587634)

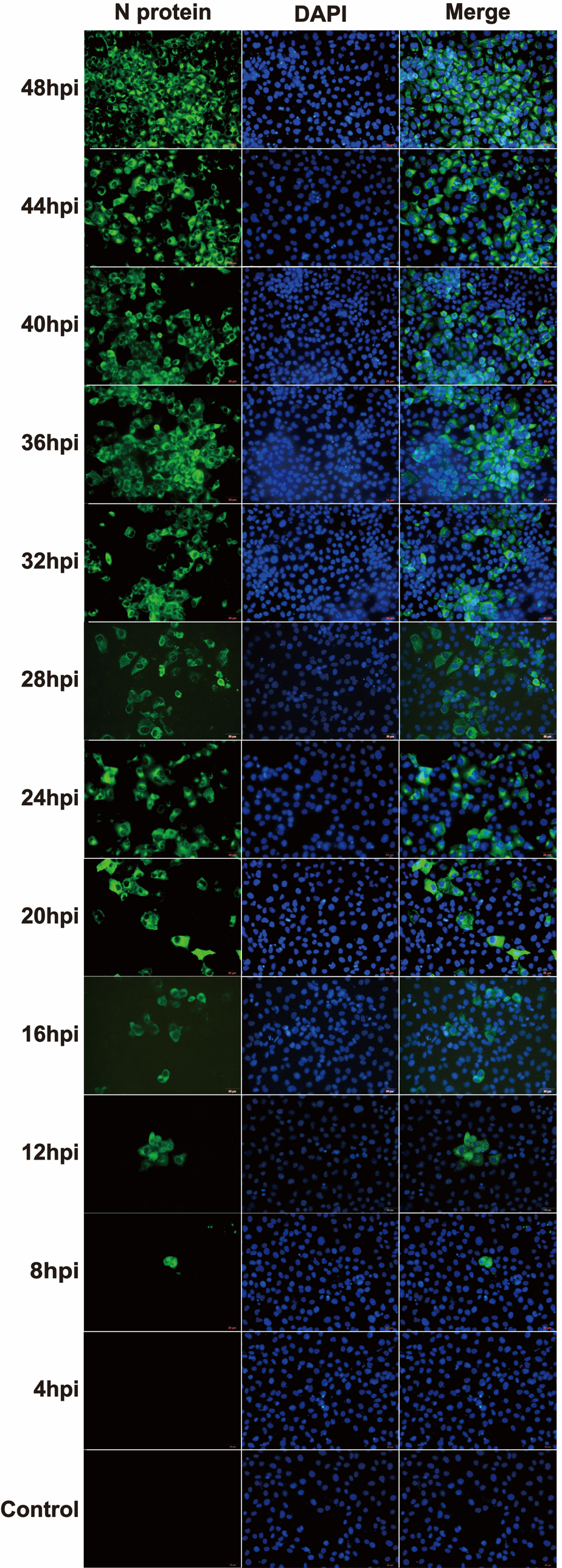

Supplement: Supplementary file 1 [file Image_1.jpeg]

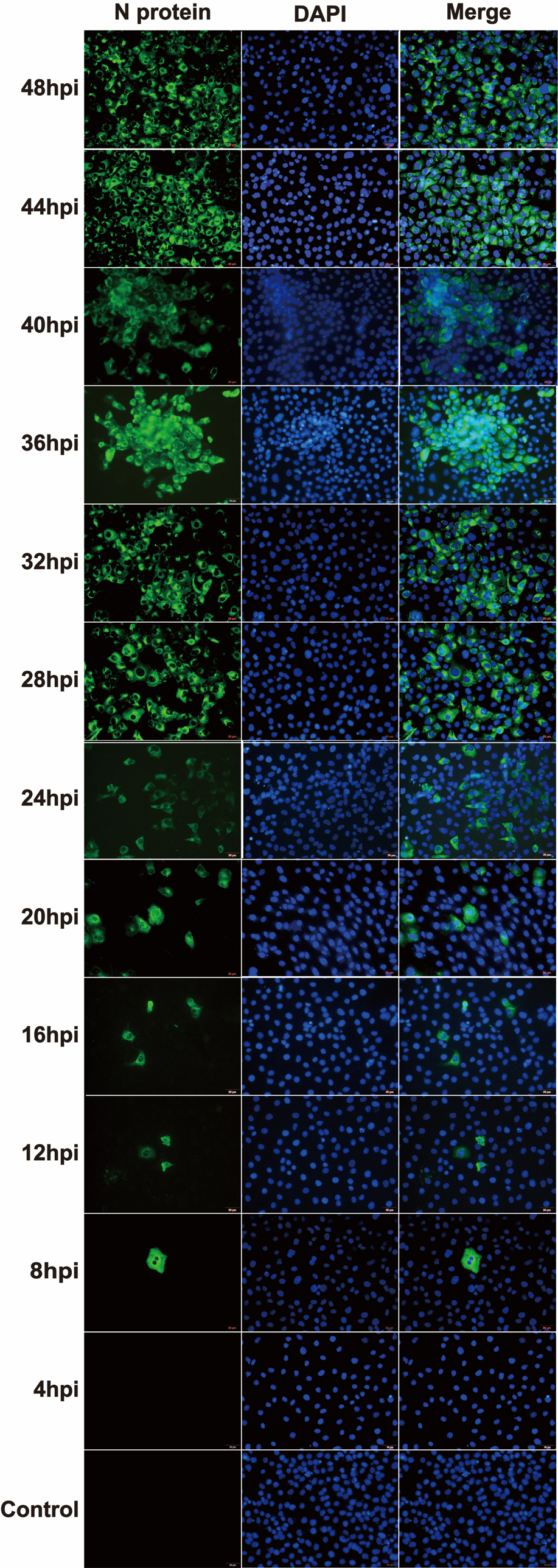

Supplement: Supplementary file 2 [file Image_2.jpeg]

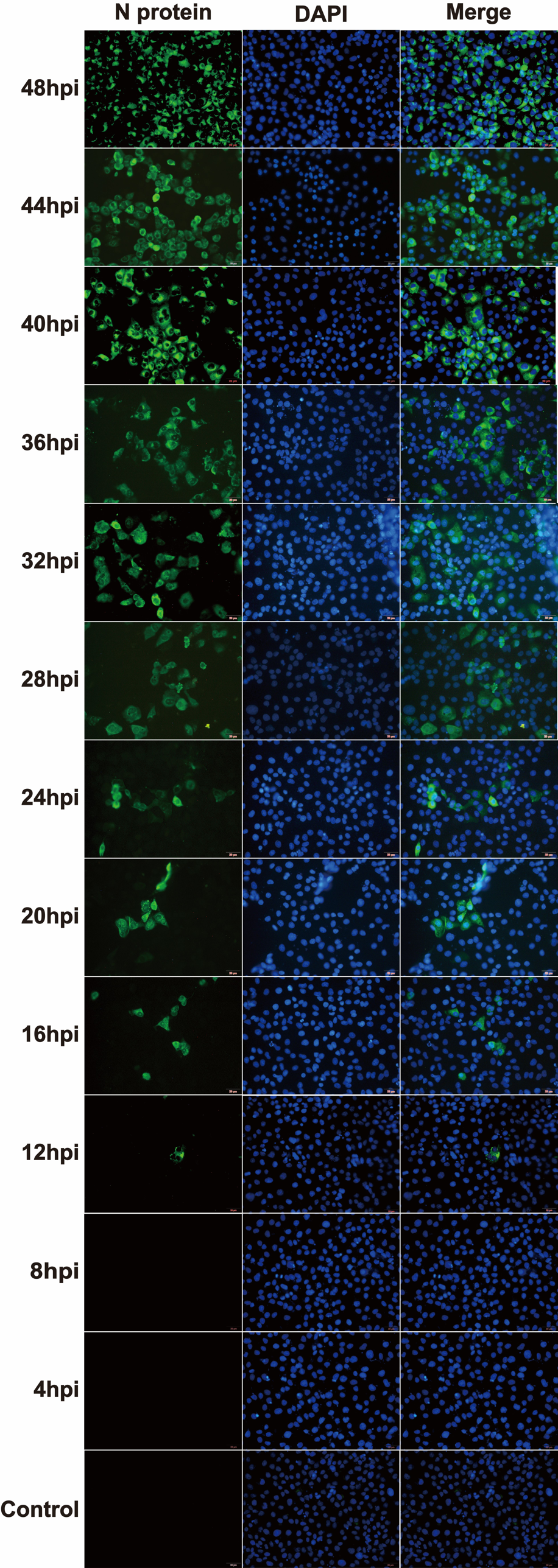

Supplement: Supplementary file 3 [file Image_3.jpeg]

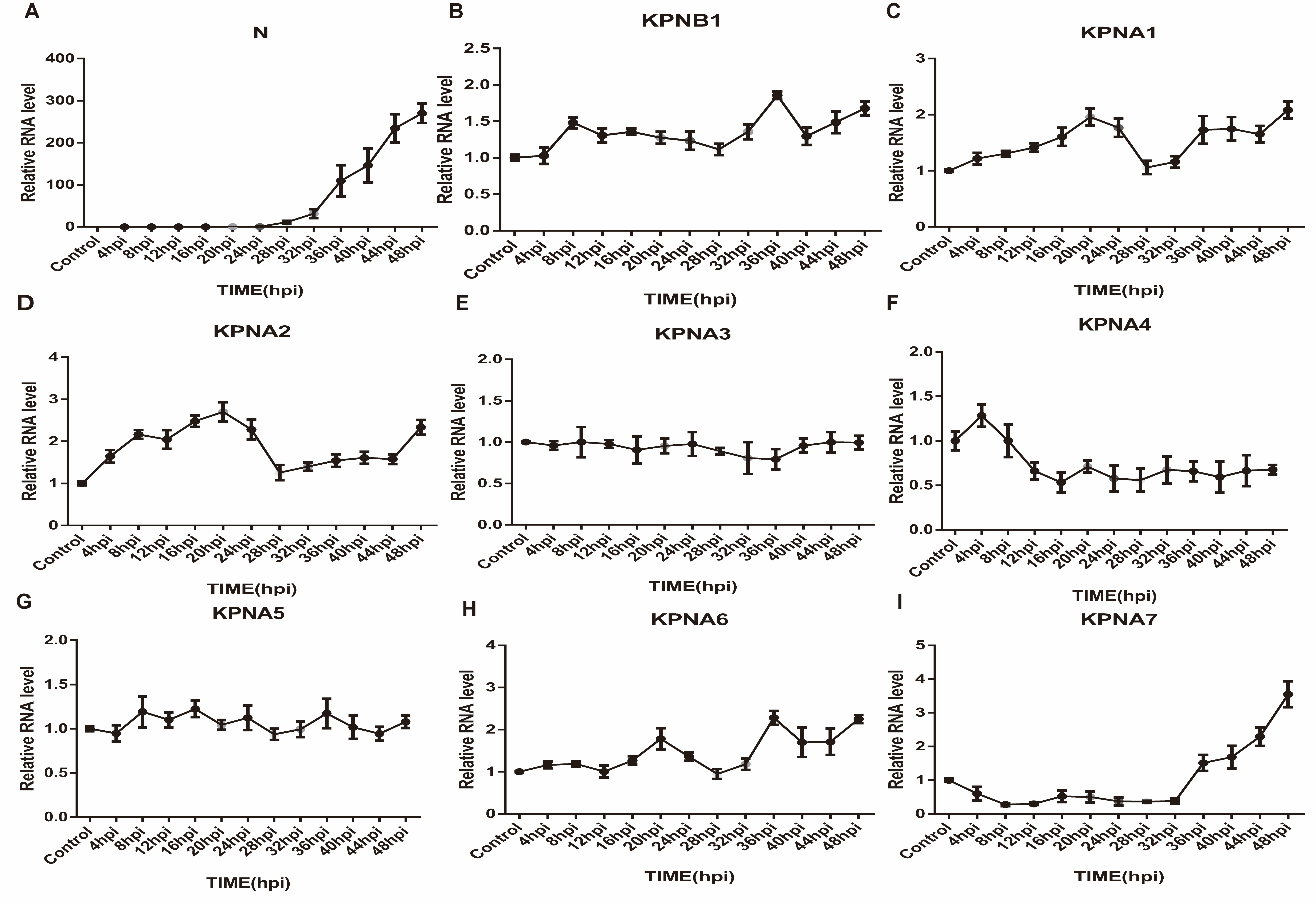

Supplement: Supplementary file 4 [file Image_4.tif]

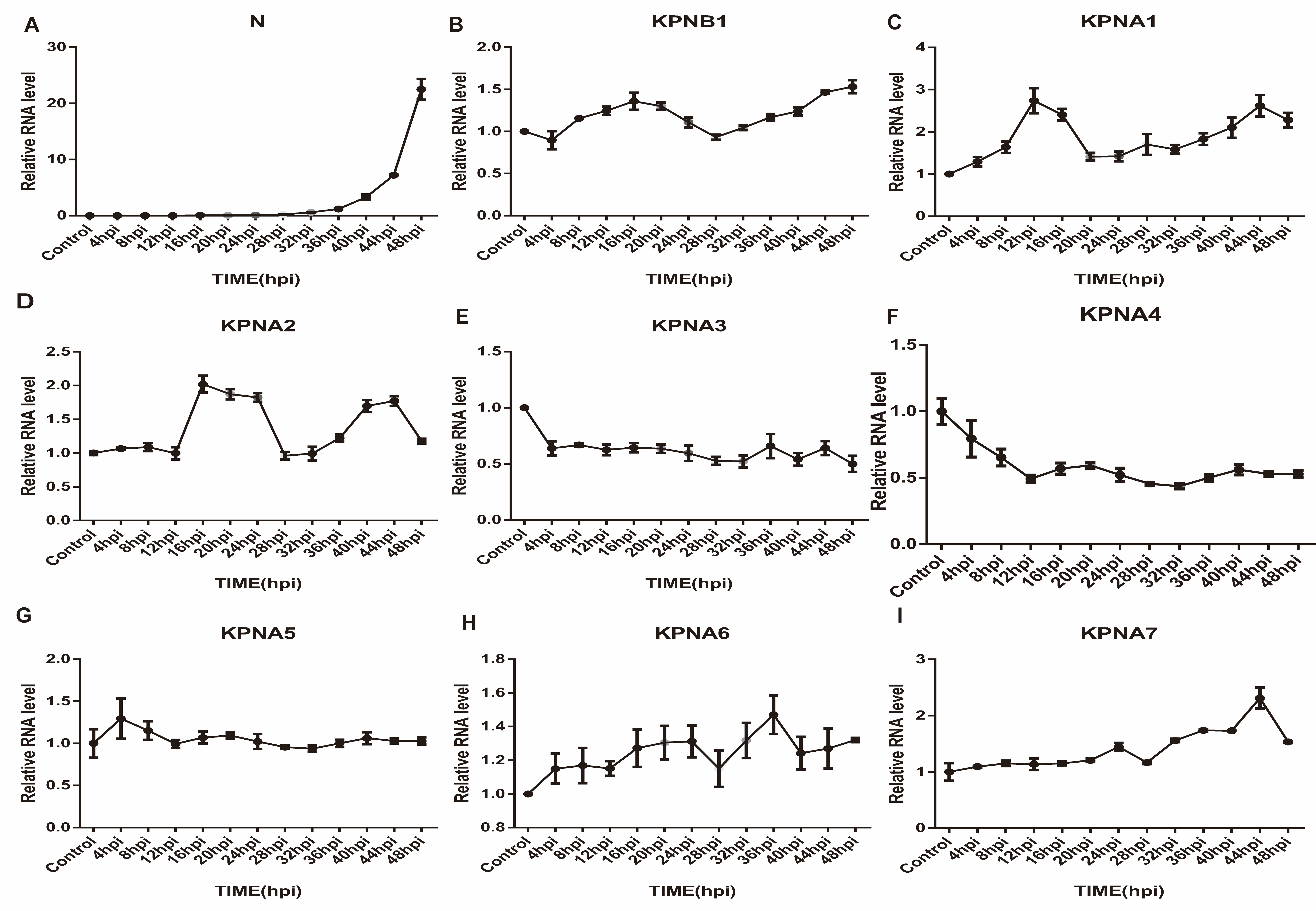

Supplement: Supplementary file 5 [file Image_5.tif]

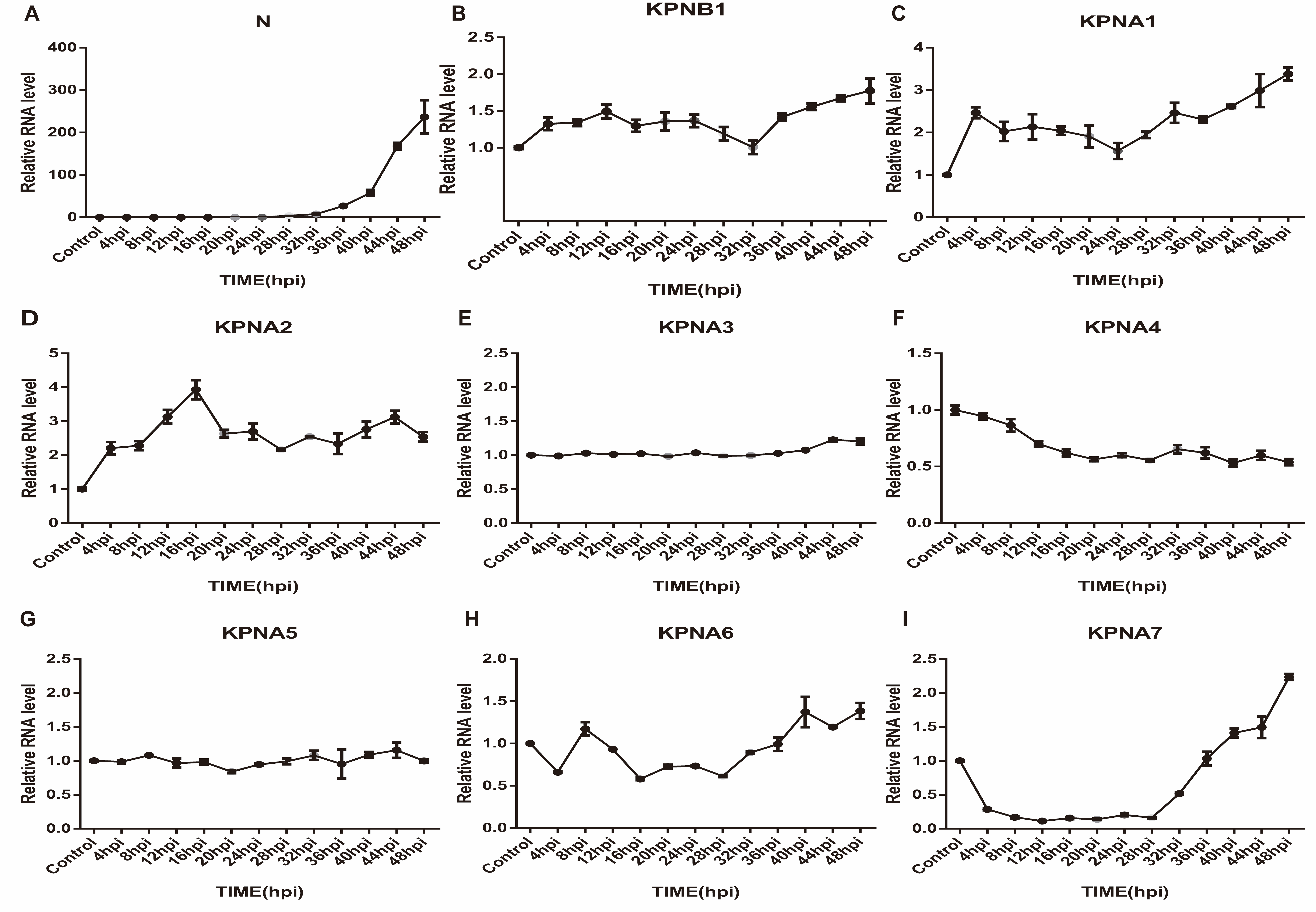

Supplement: Supplementary file 6 [file Image_6.tif]
